# Magnetically Actuated Shape Memory Polymers for On-Demand Drug Delivery

**DOI:** 10.3390/ma15207279

**Published:** 2022-10-18

**Authors:** Anand Utpal Vakil, Maryam Ramezani, Mary Beth B. Monroe

**Affiliations:** Department of Biomedical and Chemical Engineering, BioInspired Syracuse: Institute for Material and Living Systems, Syracuse University, Syracuse, NY 13244, USA

**Keywords:** shape memory polymers, polyurethanes, drug delivery, actuation, magnetism, thermoplastic, thermoset

## Abstract

Repeated use of intravenous infusions to deliver drugs can cause nerve damage, pain, and infection. There is an unmet need for a drug delivery method that administers drugs on demand for prolonged use. Here, we developed magnetically responsive shape memory polymers (SMPs) to enhance control over drug release. Iron oxide magnetic nanoparticles (mnps) were synthesized and incorporated into previously developed SMPs to enable magnetically induced shape memory effects that can be activated remotely via the application of an alternating magnetic field. These materials were tested for their shape memory properties (dynamic mechanical analysis), cytocompatibility (3T3 fibroblast viability), and tunable drug delivery rates (UV–VIS to evaluate the release of incorporated doxorubicin, 6-mercaptopurine, and/or rhodamine). All polymer composites had >75% cytocompatibility over 72 h. Altering the polymer chemistry and mnp content provided methods to tune drug release. Namely, linear polymers with higher mnp content had faster drug release. Highly cross-linked polymer networks with lower mnp content slowed drug release. Shape memory properties and polymer/drug interactions provided additional variables to tune drug delivery rates. Polymers that were fixed in a strained secondary shape had a slower release rate compared with unstrained polymers, and hydrophobic drugs were released more slowly than hydrophilic drugs. Using these design principles, a single material with gradient chemistry and dual drug loading was synthesized, which provided a unique mechanism to deliver two drugs from a single scaffold with distinct delivery profiles. This system could be employed in future work to provide controlled release of selected drug combinations with enhanced control over release as compared with previous approaches.

## 1. Introduction

Drug delivery systems can include a combination of drug carriers, manufacturing techniques, and routes to delivering a therapeutic drug to its target site to achieve the required therapeutic effect. Depending on the release mechanism, drug delivery can be categorized as targeted delivery or controlled release. Targeted delivery involves drug release at the target site without affecting any surrounding tissues [1,2]. Controlled release of drugs at a specific interval and rate is employed to achieve a sustained drug release [3]. An ideal drug delivery system ensures that the drug is available for the desired duration, above the minimal effective concentration and below the maximum tolerable concentration, while not displaying any adverse physiological effects on surrounding tissues [4].

Over 90% of hospitalized patients undergo some form of infusion therapy in which drugs are delivered intravenously [5]. While this method allows access to the entire body via the bloodstream and enables the administration of large volumes of drug infusions, there are several limitations associated with intravenous infusions, including uneven drug distribution and the inability to reach the target site at the desired dosage [6]. Intravenous administration could result in drugs reaching nontargeted tissues/organs and having adverse effects on healthy tissues. Repeated intravenous administration can cause pain at the site of injection and increases the patient’s risk of bacterial infection, requiring strict aseptic conditions at all times [7].

An alternative for controlled drug delivery includes transdermal patches [8]. This system involves the transport of drugs across the skin from either a reservoir or a matrix loaded with drugs. A major limitation of transdermal patches is that very few drug products have been developed that successfully transport across the skin due to the barrier provided by the stratum corneum of the epidermis layer of the skin [9]. This approach, therefore, requires that the drug molecule be small enough to penetrate the skin. One method to overcome these limitations involves the use of microneedle patches that penetrate the skin to allow a larger range of drugs to be administered [10]. However, microneedle patches have lower dosage accuracy compared with hypodermic needles [11].

Another approach to overcome these limitations involves the use of on-demand pulsatile drug delivery. Pulsatile drug delivery is the rapid release of a drug within a short period, followed by a specified lag time with little or no drug being released [12]. This technique requires the use of an external trigger to initiate release from a drug-loaded polymer composite at the desired location and provides control over drug delivery timing, location, and concentration. Multiple on-demand drug delivery systems have been developed that utilize an external stimulus to trigger drug release. Chunder et al. developed a pH-responsive scaffold by electrospinning poly(acrylic acid) and poly(allylamine hydrochloride) [13]. Methylene blue was used as a model cationic drug that was released at low pH. This system requires that drugs be cationic or anionic and involves control over pH, which limits its applicability.

Light-triggered on-demand drug release from an implanted depot was developed by Carling et al. [14]. This approach utilizes a biphenyl derivative that is photocleaved by blue light irradiation to release a hydrophobic cargo, dexamethasone. While this system shows promise in subcutaneous implants, light-triggered systems could have limited applicability in deep implantation sites. Magnetic nanoparticles have been explored for over 30 years for use in intravenous drug delivery [15,16]. Their incorporation into drug delivery scaffolds could enable magnetically controlled release throughout the body. To that end, Satarkar et al. developed magnetic hydrogel nanocomposites that can be actuated remotely to trigger on-demand drug release [17]. In that work, N-isopropyl acrylamide hydrogels were loaded with magnetic nanoparticles and exposed to high-frequency alternating magnetic fields to enable the release of vitamin B12 over time. An increase in drug release was observed upon application of the alternating magnetic field, and drug release was controlled upon exposure to the alternating magnetic field every hour over 12 h, with 10 min of magnetic actuation at each time point. In another approach, Zhao et al. employed iron oxide nanoparticles to cross-link poly(vinyl alcohol) gel beads [18]. In this system, the application of a magnetic field induced nanoparticle densification to prevent drug release, which has been observed by others [19]. They observed some minor differences in drug release based on the application of a magnetic field to the beads.

The magnetically responsive approach is promising, but more robust control over drug delivery could be attained by using shape memory polymers (SMPs) whose shape change can be triggered remotely. SMPs are smart materials that are prepared in an original shape and temporarily deformed and stored in a secondary shape after exposure to an external stimulus. The original shape can be recovered upon exposure to a second external stimulus. The shape recovery of SMPs can be actuated by a range of triggers, including temperature, pH, light, and electrical or magnetic impulse [20,21,22,23,24]. Polydiocitrate-based shape memory elastomers with drug-releasing capabilities were developed by Serrano et al. [25]. Hydrophobic microdomains within the polymer were used as reservoirs to entrap and subsequently release hydrophobic drugs. Exposing the polymers to PBS at varying temperatures (above and below their transition temperature) altered the drug release from the polymer composite, thus providing a thermally induced drug release over 700 h. However, thermal actuation of SMPs is limited to temperatures below ~45 °C in the body to minimize potential thermal damage to cells.

To combine the benefits of prior magnetically responsive and SMP approaches, we hypothesized that magnetically responsive polyurethane-based SMPs could enhance controlled drug delivery. To that end, we selected SMP compositions based on previously developed thermally actuated SMP systems [26,27]. Using these materials, magnetically responsive polymer composites were prepared by incorporating iron oxide magnetic nanoparticles (mnps) into the SMPs. These materials were initially prepared with constant drug loading across the polymer and thermally fixed in a strained temporary shape that limits drug diffusion. Doxorubicin hydrochloride (a relatively hydrophilic chemotherapeutic with a molar mass of 543.5 g·mol^−1^) and 6-mercaptopurine (a relatively hydrophobic immunosuppressant with a molar mass of 152.2 g·mol^−1^) were selected for these studies, since these drugs may have reduced side effects on patients if localized and controlled release can be obtained. Rhodamine (a hydrophilic fluorescent molecule with a molar mass of 479.02 g·mol^−1^) was employed as an additional model drug due to its ease of measurement. Drug release was initiated upon mnp excitation via exposure to an alternating magnetic field. Variation in shape recovery rates and subsequent drug release was controlled by altering the polymer chemistry and mnp concentration. Control over drug release within a single implant was achieved by varying the mnp concentration, thus allowing controlled drug release from specified regions of the polymer over multiple triggers. These polymer composites could combine the previously observed benefits of magnetically responsive drug delivery with those of SMP-based drug delivery scaffolds. This system provides unique control over drug release, which enables delivery of a single drug at multiple time points and/or dual drug release from the same implant at a specified location with reduced potential risks to surrounding tissues/organs.

## 2. Materials and Methods

### 2.1. Materials

All materials were purchased from Fisher Scientific (Waltham, MA, USA) as reagent grade. Hexamethylene diisocyanate (HDI), sodium borohydride, ferric chloride hexahydrate, phosphate-buffered saline (PBS), dimethyl sulfoxide (DMSO), doxorubicin hydrochloride (Dox), 6-mercaptopurine (6-MP), rhodamine B (Rhod), dibutyl(tin) dilaurate (DBTDL), Sylgard-184 poly(dimethyl siloxane) (PDMS), hydrogen peroxide (H_2_O_2_, 30%), and methanol were used as purchased. Triethylene glycol (TEG), polypropylene glycol (MW: 2000 Da, (PPG)), and N′, N′, N, N-tetrakis-2-hydroxypropyl ethylenediamine (HPED) were dried in a vacuum oven at −30 inches Hg vacuum and 40 °C overnight before use.

### 2.2. Synthesis

#### 2.2.1. Magnetic Nanoparticles

A ferric chloride (FeCl_3_.6H_2_O) solution (0.1 M in deionized water) was added dropwise to sodium borohydride (NaBH_4_, 2.5 M in deionized water) in a beaker at a ratio of 4:1 (FeCl_3_:NaBH_4_). The solutions were mixed constantly using a magnetic stir plate (Fisher Scientific, Waltham, MA, USA) at 1050 rpm. The reaction produced bubbles and was therefore allowed to continue until no more bubble formation was observed (~1 h). Magnetic nanoparticles were allowed to settle to the bottom of the beaker and then washed twice with deionized water and twice with methanol. Particles were centrifuged (Sorvall X4F Pro-MD, Thermo Scientific, Waltham, MA, USA) at 10,000× *g* for 10 min at room temperature during each washing step. Washed particles were dried at 50 °C overnight, and any clusters were crushed using a glass rod. Particle size was determined via dynamic light scattering using a Zetasizer Ultra (Malvern Panalytical, Westborough, MA, USA). Approximately 1 mg of particles was added to 5 mL DI water in a disposable 10 × 10 polystyrene cell and sonicated for 5 min before testing. A graph of intensity (%) vs. particle size (d, nm) was analyzed to confirm the particle size.

#### 2.2.2. Polymers

**Figure 1:**
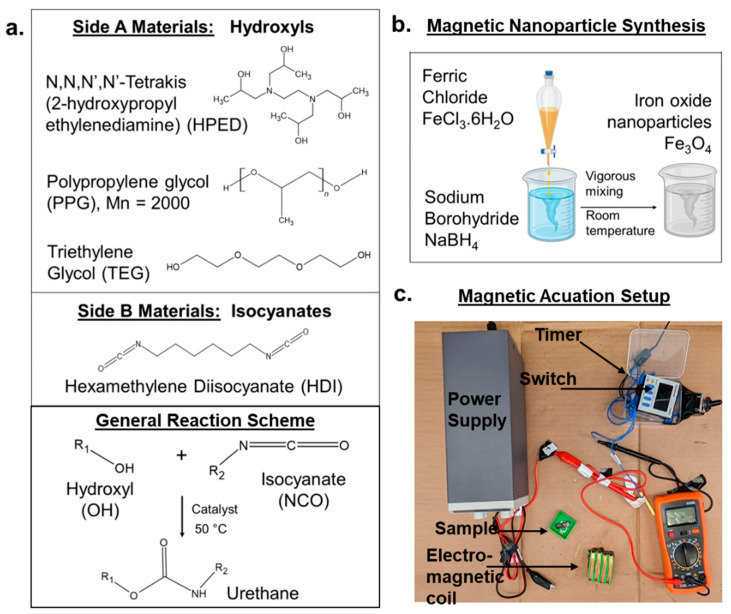
(**a**) Polymer component structures and overview of the synthesis of polyurethane SMP. (**b**) Overview of the synthesis of magnetic nanoparticles. (**c**) Magnetic circuit setup employed for magnetic actuation of SMPs.

All synthesis was performed in a glove box (Labconco, Kansas City, MO, USA) while maintaining a dry inert atmosphere using nitrogen passed through a drying train (Labconco, Kansas City, MO, USA) to maintain relative humidity below 200 ppm. The composition of each formulation is shown in Table 1, and component structures are shown in Figure 1a. All compositions included HDI as the isocyanate component, and a combination of PPG, TEG, and/or HPED was employed as the hydroxyl component to form polyurethanes. First, the drug/dye (Dox, 6-MP, Rhod) and mnps were weighed out into a speed mixer cup, shown in Table 1. In short, 5 mg of drug/dye was added to each test sample (10 g), and either 50 mg or 100 mg of mnps was added to each sample (10 g). Then, the monomers and catalyst (DBTDL) were added and mixed in a speed mixer (FlackTek, Landrum, SC, USA) at 3500 rpm. The duration of mixing varied depending on the reactivity of each component, as shown in Table 1. The monomers were then allowed to react in an oven in the speed mixer cup at 50 °C for 48 h. 

Formulations with dual compositions were prepared in a two-step process. The components for PPG TEG were mixed in a speed mixer and then poured immediately onto a petri dish lined with a Teflon liner. One-half of the petri dish was blocked using a PDMS mold to prevent the reaction components from spreading across the entire petri dish. The petri dish was immediately placed in an airtight container in an oven at 50 °C for 48 h. The PDMS mold was then peeled off, and the HPED TEG mixture was mixed and poured onto the remaining half of the petri dish while leaving the PPG TEG side intact. This mixture was then allowed to react for another 48 h at 50 °C to form a dual SMP with PPG TEG on one side and HPED TEG on the other side.

### 2.3. Hydrophobicity

Thin slices (2 mm thick, 1.5 cm wide, and 6.5 cm long) were cut from each formulation to measure the contact angle using a goniometer (Model 500, Ramé-hart Co., Succasunna, NJ, USA). Water droplets (0.2 mL, *n* = 3) were placed onto the films, and 100 images were captured at a 0.01 s interval using a SuperSpeed U4 series camera. Each image was analyzed using the DROPimage software to determine the angle between the water droplet and the material surface. An average of 100 measurements was used to determine the contact angle for each water droplet.

### 2.4. Mechanical Properties

Tensile testing was performed using a 24 N load cell (Test Resources, Shakopee, MN, USA). Samples (*n* = 3) were cut in a dogbone shape (ASTM D638 Type IV scaled down by a factor of 4) with a gauge length of 6.25 mm and a width of 1.5 mm. The thickness of each sample was measured using calipers. Samples were subjected to a tensile force at a rate of 2 mm/min until failure to measure the elastic modulus, ultimate tensile strength, and elongation at break. The measurements were carried out on dry and wet samples. To prepare wet samples, specimens were placed in water at 50 °C for 4 h before testing.

### 2.5. Thermal Characterization

A differential scanning calorimeter (DSC Q200, TA Instruments, New Castle, DE, USA) was used to measure the glass transition and melting temperatures of polymers. Dry samples (*n* = 3) were weighed (~3–5 mg) and placed in T_zero_ aluminum pans to be tested. Samples were cooled to −40 °C at 10 °C/min, kept isothermally for 2 min, heated to 120 °C at 10 °C/min, kept isothermally for 2 min, cooled to 50 °C at 2 °C/min, kept isothermally for 20 min, cooled to −40 °C at 2 °C/min, kept isothermally for 2 min, and then heated back to 120 °C at 10 °C/min. Transition temperatures were measured during the second heating cycle. To test samples in wet conditions, thin slices (*n* = 3) were placed in DI water at 50 °C for 4 h, patted dry, cut to weigh ~3 to 5 mg, and placed in T_zero_ aluminum pans with hermetic lids. To measure the transition temperatures, samples were cooled to −40 °C at 10 °C/min and heated to 120 °C at 10 °C/min. The transition temperatures were measured in a single heating cycle.

### 2.6. Shape Memory Properties

Samples (*n* = 3) were cut into dogbone shapes (ASTM D638 Type IV scaled down by a factor of 4) with a gauge length of 6.25 mm and a width of 1.5 mm. The width of each sample was measured using calipers before testing. Shape memory tests were performed using a dynamic mechanical analyzer (DMA Q800, TA Instruments, New Castle, DE, USA). The samples were heated to 80 °C at 2 °C/min and held isothermally for 2 min. Then a controlled force was applied at 0.03 N/min to a maximum limit of 18 N until the sample reached 20% strain. Samples were then cooled to −5 °C and held isothermally for 2 min to ensure shape fixing. The load was released at 0.03 N/min, and samples were heated back to 80 °C at 2 °C/min to measure shape recovery. This procedure was repeated thrice for each sample. Shape fixity (*R_f_*) and shape recovery (*R_r_*) were measured in each cycle (*N)* according to Equations (1) and (2), respectively, where *ε_m_* is the maximum strain at loading, *ε_u_* is strain after unloading (fixed shape), and *ε_p_* is the remaining strain after recovery (permanent strain).

(1) $$R_{r}\left(N\right)=\frac{\epsilon_{m}-\epsilon_{p}\left(N\right)}{\epsilon_{m}-\epsilon_{p}\left(N-1\right)}$$

(2) $$R_{f}\left(N\right)=\epsilon_{u}/\epsilon_{m}$$

### 2.7. Cytocompatibility

Samples (*n* = 3) that were not loaded with drugs were cut using a 6 mm biopsy punch (Fisher Scientific, Waltham, MA, USA), placed in 15 mL DI water, and sonicated (CPX Digital Series Ultrasonic bath, Fisher Scientific, Waltham, MA, USA) constantly over 1 week. The surrounding solution was changed every 2 h during the first 2 days and then changed twice over the remaining 5 days. On the last day, samples were placed in sterile PBS and sonicated overnight. Samples were then sterilized via UV-C radiation (UV sterilizer and sanitizer cabinet, Skin Act, Pacoima, CA, USA) for 3 h. NIH/3T3 Swiss mouse fibroblasts (ATCC-CCL92, Manassas, VA, USA, passage 6) were seeded onto 24-well plates at a density of 10,000 cells/well and incubated for 24 h. The cells were cultured with Dulbecco’s Modified Eagle Medium supplemented with 10% heat-inactivated fetal bovine serum and 1% penicillin–streptomycin (P/S, Gibco, Thermo Scientific, Waltham, MA, USA). Cell morphology was assessed using a Zeiss Axiovert inverted microscope to confirm uniform cell distribution. Sterilized samples were placed in 0.4 µm Transwell^®^ inserts in a 24-well cell culture plate to measure indirect cytocompatibility at 3, 24, and 72 h using a resazurin cell viability assay. At each time point, samples and Transwell^®^ were removed from the plate. The media in each well were replaced with resazurin stain after rinsing with sterile PBS. Fluorescence from cells was measured using a plate reader (FLx800, BioTek Instruments Inc., Winooski, VT, USA) at 570 nm. Cytocompatibility was assessed according to Equation (3). Cells (*n* = 3) not exposed to any samples or Transwell^®^ were used as positive cytocompatible controls, and cells exposed to 20 µL of 30% H_2_O_2_ were used as negative cytotoxic controls.

(3) $$\mathrm{Cytocompatibility}\;\left(\%\right)=\frac{\mathrm{abs}570\left(x\right)\;–\;\mathrm{abs}570\left(\mathrm{negative}\right)}{\mathrm{abs}570\left(\mathrm{positive}\right)\;–\;\mathrm{abs}570\left(\mathrm{negative}\right)}\times 100$$

### 2.8. Drug Release

#### 2.8.1. Magnetic Circuit

The magnetic coil consisted of 200 turns of 36 AWG magnet winding wire, wound across four coils (3D-printed acrylonitrile butadiene styrene, 5 cm long, 2 cm wide) in parallel to generate a maximum magnetic field strength of 5 mT. A relay switch was used along with a digital timer (Panasonic LT4H-DC24V) to generate an alternating magnetic field at a frequency of 5 Hz. The main power supply was maintained at a constant 12 V. The setup of the circuit is shown in Figure 1b. Polymers were subjected to the alternating field by placing the samples in a reservoir (3D-printed, 10 mL volume) within the coils for 10 min with an alternating magnetic field strength of 0.5 mT and 5 Hz frequency.

#### 2.8.2. Drug Release Measurements

Samples (*n* = 3) were placed in 6 mL PBS in 20 mL scintillation vials at 37 °C. At set time points and/or after exposure to the magnetic field, the surrounding solution was aliquoted and mixed with DMSO (2X dilution) before evaluation via UV–VIS light spectroscopy (Evolution 60, Thermo Scientific, Waltham, MA, USA). Drug/dye concentrations were quantified using reference peaks (rhodamine B: 555 nm, 6-MP: 333 nm, Dox: 480 nm) to assess release over time. Samples containing single polymer composition and a single drug were either subjected to an alternating magnetic field for 40 min or placed in an oven at 37 °C for 40 min to compare drug release with and without exposure to a magnetic field. Samples containing dual drug combinations were subjected to an alternating magnetic field for 10 min at each time point (1, 4, and 7 h) to initiate shape change and subsequent drug release. To compare release rates between strained and unstrained samples, different amounts of strains were applied to samples depending on their stretchability (assessed based on their elastic deformation range during tensile testing). HPED TEG and HPED PPG samples were stretched at 20% strain, while PPG TEG was stretched to 40% strain.

### 2.9. Statistics

ANOVA with Tukey’s post hoc was used to compare sample measurements. Statistical significance was taken as *p* < 0.05.

## 3. Results and Discussion

### 3.1. Hydrophobicity

**Figure 2:**
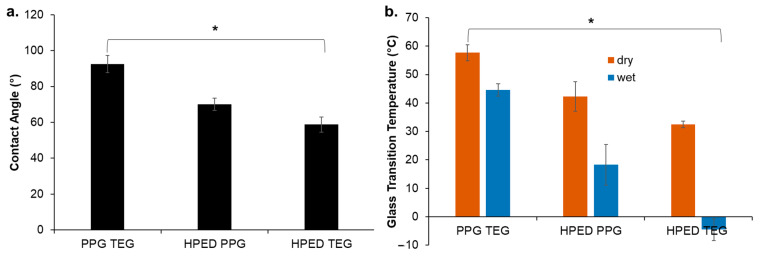
(**a**) Contact angle and (**b**) glass transition temperature in dry and wet conditions. Mean ± standard deviation displayed. *n* = 3. * *p* < 0.05 between all formulations and measurement conditions.

PPG TEG had the highest water contact angle of 92°, followed by HPED PPG at 69° and HPED TEG at 58°, as seen in Figure 2a. An increase in hydrophobicity was observed among the samples with PPG in the polymer network, and PPG incorporation resulted in a waxy outer layer that helped to repel water and delay polymer wetting. In general, the major factors that contribute to increased hydrophobicity are network homogeneity and longer carbon chains [28]. PPG has a relatively higher carbon content compared with the other hydroxyl-containing monomers shown in Figure 1a. The PPG TEG formulation is also a linear polymer (vs. cross-linked networks that form with HPED polyols), which enables increased component mobility during polymerization and likely results in higher network homogeneity to further contribute to increased hydrophobicity.

### 3.2. Thermal Characterization

A dry glass transition temperature above room temperature would enable stable storage of polymers in their temporary/deformed state during regular storage. A wet glass transition temperature above 37 °C would prevent deformed SMPs from changing shape after implantation unless they have been exposed to a localized increase in temperature (i.e., upon exposure to the magnetic field). Thus, glass transition temperatures were measured in wet and dry conditions for the synthesized polymers (Figure 2b). All polymers had dry glass transition temperatures above room temperature. The PPG TEG polymer had the highest wet glass transition temperature at 44 °C, which is attributed to its increased hydrophobicity. Thus, this polymer has the potential to stably stay in its secondary shape after implantation. In general, wet glass transition temperature trends followed contact angle measurements, with HPED TEG having the lowest wet glass transition temperature due to its increased hydrophilicity (Figure 2b).

### 3.3. Mechanical Properties

A lower elastic modulus and higher elongation at break enable polymer deformation and fixing in the secondary shape. PPG TEG had the lowest elastic modulus of 80 kPa and the highest elongation at a break of 680% in dry conditions (Table 2). HPED TEG had the highest elastic modulus of 595 kPa, the highest ultimate tensile strength of 29,200 kPa, and lower elongation at a break of 170%. The highly cross-linked network of HPED TEG provides the stiffest polymer, while linear PPG TEG is less stiff, which enables higher elongation at break. It was observed that HPED PPG and HPED TEG formulations underwent plastic deformation upon stretching beyond 40% strain, which limits the strain that can be applied during shape fixing.

### 3.4. Shape Memory Properties

**Figure 3:**
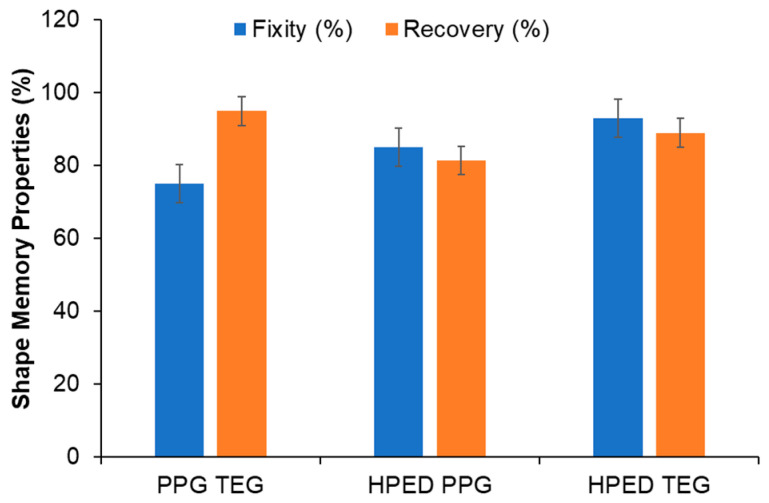
Shape memory properties are expressed in terms of shape fixity and recovery. Mean ± standard deviation displayed. *n* = 3. No statistical differences were observed between formulations.

All formulations had shape fixity and recovery above 75% (Figure 3). This property enables stable fixing of the temporary shape and recovery of the primary shape upon exposure to a stimulus (i.e., alternating magnetic field). During the tests, all materials were stretched to a maximum of 40% strain to avoid plastic deformation. HPED TEG and HPED PPG formulations utilize net points provided by hydrogen bonding between urethane groups at cross-linking sites to fix their shape. The urethane net points of the PPG TEG formulation are found in the hard segments provided by TEG and HDI, which also rely upon hydrogen bonding to fix their shape. While no significant differences were observed between shape memory properties, PPG TEG had slightly lower shape fixity than the other two formulations. This result could be attributed to the chain mobility restrictions provided by PPG soft segments in this linear polymer.

### 3.5. Cytocompatibility

**Figure 4:**
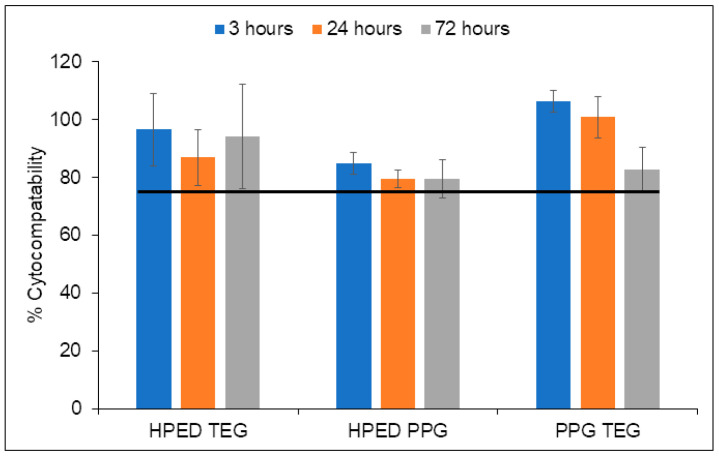
Cytocompatibility of 3T3 mouse fibroblasts over 72 h in the presence of HPED TEG, HPED PPG, and PPG TEG loaded with 100 mg of mnp/8 g of polymer. Mean ± standard deviation displayed. *n* = 3. The horizontal line denotes ISO 10993-5 standard (75% cytocompatibility).

All formulations containing the higher concentration of mnp (100 mg mnp/8 g polymer) had cytocompatibility above 75% over 72 h (Figure 4). This result shows that these magnetically responsive materials are cytocompatible according to ISO 10993-5 standards [29].

### 3.6. Magnetic Particles

The size of the paramagnetic Fe_3_O_4_ particles was determined via dynamic light scattering. The average hydrodynamic particle diameter was found to be close to 100 nm, thus confirming the formation of mnps with consistent size. Future work will include more in-depth characterization of nanoparticles to enable finer tuning of magnetic responsiveness.

### 3.7. Drug Release

Initially, drug release was measured to study the effects of different parameters on release profiles. All release profiles shown were obtained using doxorubicin hydrochloride as the sample drug unless specified otherwise.

#### 3.7.1. Effects of Polymer Formulation

**Figure 5:**
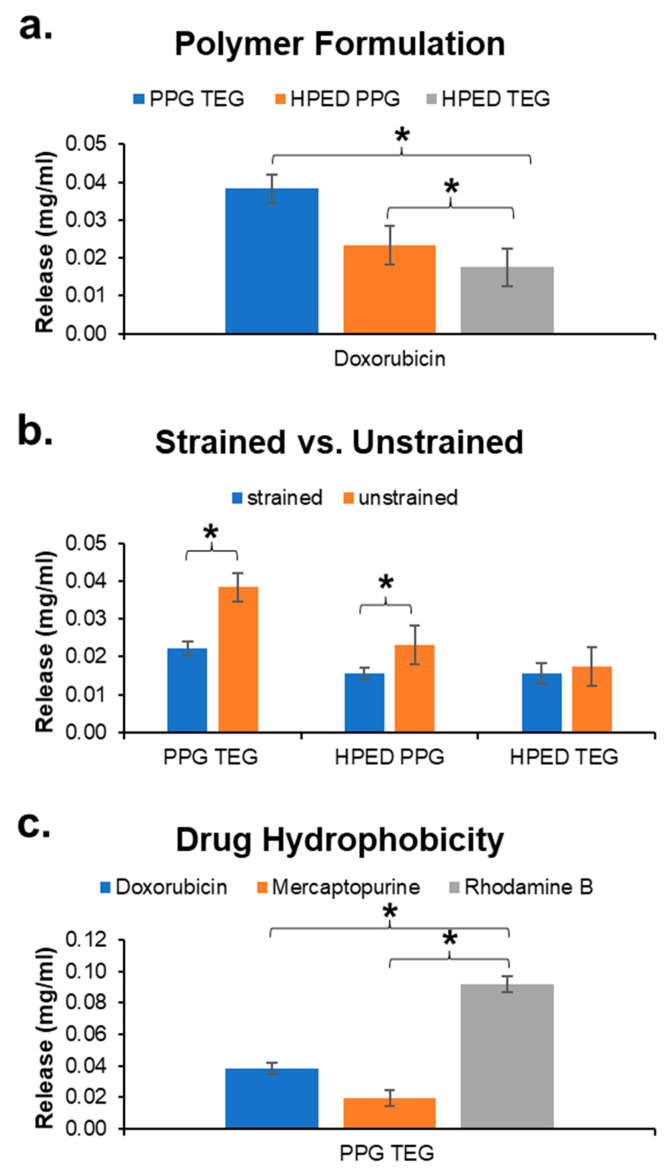
Drug release is based on the physical and chemical properties of polymers and drugs. (**a**) Effect of polymer formulation, (**b**) effect of straining/fixing samples in secondary shapes, and (**c**) effect of drug hydrophobicity. Mean ± standard deviation displayed. *n* = 3. * *p* < 0.05 between formulations under brackets.

In unstrained samples (containing 100 mg mnp per 10 g sample) with the application of a magnetic field, it was observed that Dox release rates from PPG TEG were fastest compared with those from HPED PPG and HPED TEG (Figure 5a). This result can be attributed to the linear network of PPG TEG compared with the cross-linked networks of HPED PPG and HPED TEG. A highly cross-linked network can physically trap the drugs within the structure, thus limiting drug diffusion to the surrounding medium.

#### 3.7.2. Effects of Applied Strain/Shape Fixing

Dox release was consistently reduced across all formulations when the polymers were strained and fixed in a secondary shape compared with unstrained polymers in their primary shape (Figure 5b). By straining the samples, the polymer chains are brought closer together, thus creating a barrier to drug diffusion out of the network. Since PPG TEG can be strained to higher amounts without plastic deformation, the difference in release rates between strained (40% strain) and unstrained conditions was the highest for this formulation. Additionally, the high wet glass transition temperature of PPG TEG samples resulted in minimized shape recovery during testing. Due to the limited stretchability of HPED PPG and HPED TEG films (18% to 20% strain applied here), the differences in release profiles before and after straining these materials were smaller, with no significant difference observed in HPED TEG samples. These results also correlate with wet glass transition temperature measurements, indicating that passive shape recovery after plasticization in 37 °C water plays a role in this process as well.

#### 3.7.3. Effects of Drug Hydrophobicity

Drug release rates were also influenced by drug hydrophobicity (Figure 5c). Due to the limited water solubility of Dox and 6-MP, release was carried out in PBS, and then the solutions were diluted with DMSO in a 1:1 ratio to measure the released drug concentration. It was observed that Rhod, which has a higher solubility in water of 15 mg/mL [30], had the highest release rate from PPG TEG, followed by Dox with a water solubility of 10 mg/mL and 6-MP with the lowest solubility of 0.5 mg/mL in a 1:1 combination of DMSO and PBS [30].

#### 3.7.4. Effect of a Magnetic Field

**Figure 6:**
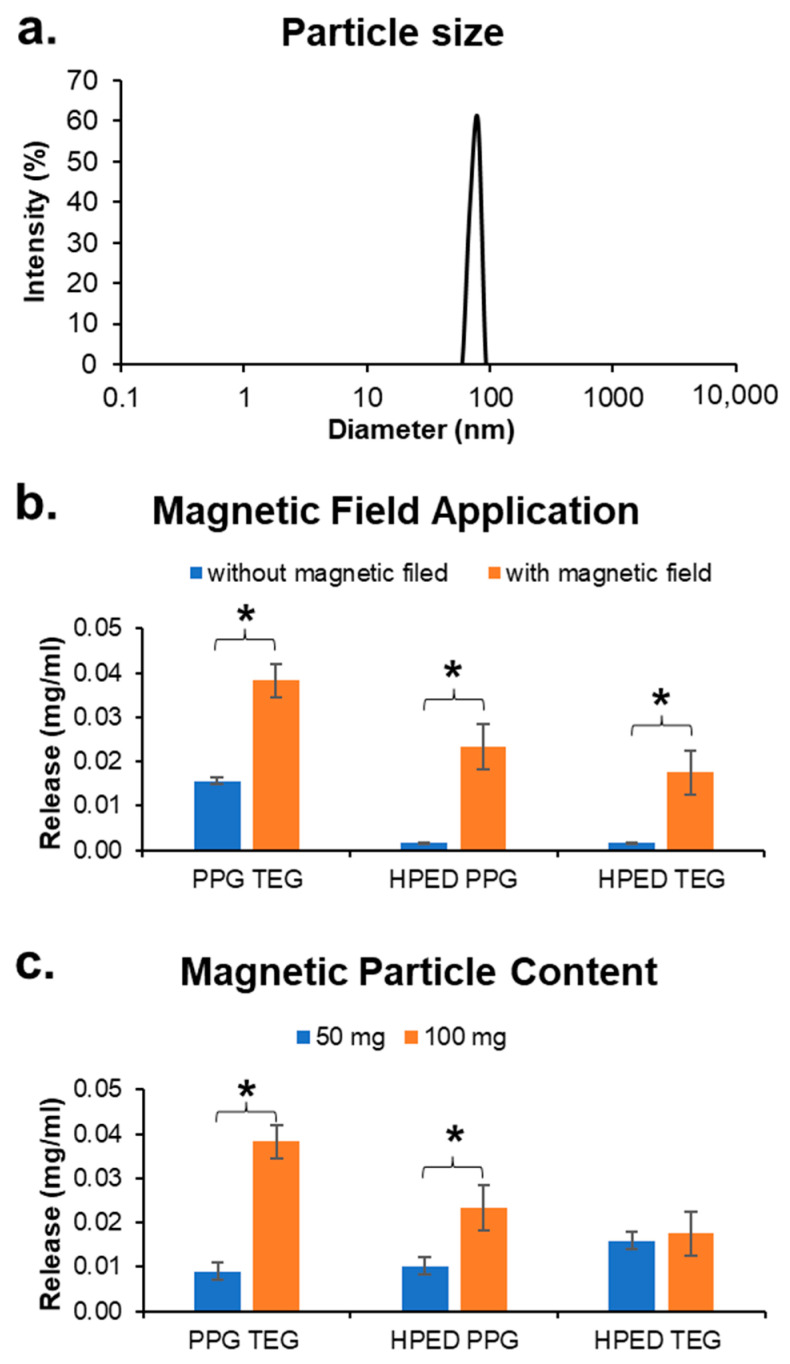
Drug release based on magnetic field application. (**a**) Average magnetic nanoparticle size (*n* = 5), (**b**) effect of a magnetic field, and (**c**) effect of magnetic particle content on Dox release. Mean ± standard deviation displayed. *n* = 3. * *p* < 0.05 between formulations under brackets.

The release of Dox from unstrained polymers was significantly influenced by exposure to an alternating magnetic field (Figure 6b). Release concentrations were obtained after exposing drug-loaded polymers to an alternating magnetic field for 40 min. During this time interval, there was a local increase in the polymer temperature that triggered drug release. The temperature of the surrounding solution was measured using an infrared thermometer to ensure that heat radiated by the magnetic coil and/or the mnp excitation did not transfer to the surroundings. The surrounding solution temperature was ≤40 °C during the application of the magnetic field, which should not be harmful to the surrounding cells or tissues. It is hypothesized that exposure to the alternating magnetic field excites the magnetic particles to switch magnetic orientations (north and south) at a frequency of 0.5 Hz. This excitation induces vibrations among particles, which result in a localized increase in temperature in the polymers to soften polymer chains and increase drug diffusion out of the network.

#### 3.7.5. Effect of Mnp Concentration

Tuning mnp concentrations can be used as an additional mechanism to control release. Lower mnp concentrations result in lower localized temperature increases due to smaller vibrations caused by the particles. Thus, reducing mnp concentration reduced drug release during exposure to a magnetic field at the same strength and frequency compared with polymers with higher mnp content (Figure 6c). The difference in release rates among HPED TEG with varied mnp content was lower. This result may be due to increased network cross-link density, which limits drug diffusion out of the polymer and/or due to the low wet glass transition temperature, which minimizes the effects of the magnetic field on the already-plasticized polymer network.

### 3.8. Dual Drug Release

**Figure 7:**
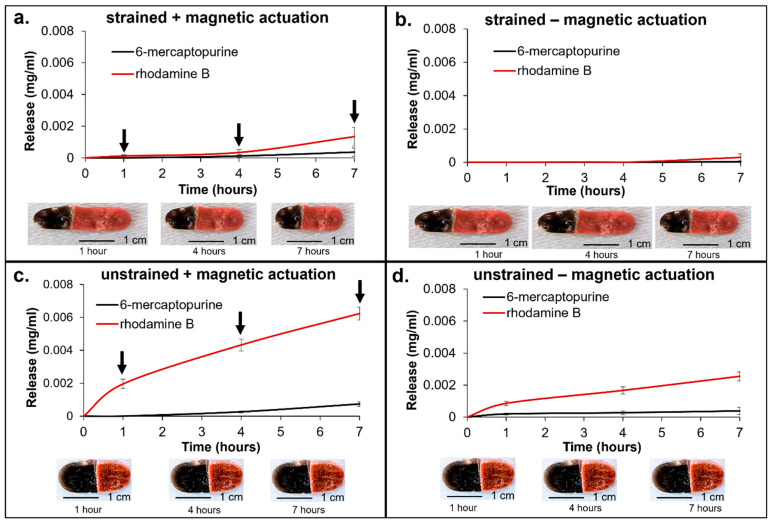
Dual drug release from a single scaffold. Rhodamine and 6-MP were released from strained polymer samples (**a**) under magnetic actuation and (**b**) without magnetic actuation. Rhodamine and 6-MP release rates were measured from the same unstrained samples (**c**) under magnetic actuation and (**d**) without magnetic actuation. The downward arrow indicates time points at which polymers were exposed to an alternating magnetic field for 10 min (**a**,**c**) and tested for drug release. Images depicting a change in polymer dimensions (i.e., shape recovery) upon each magnetic field application are presented in (**a**,**b**), while images in (**c**,**d**) show that unstrained samples did not change shape during testing. Mean ± standard deviation displayed. *n* = 3.

After comparing the release profiles from individual formulations, a scaffold was prepared to achieve a dual release profile of two different drugs (6-MP and Rhod) from a single polymer composite with varying polymer composition and mnp loading. Films were tested either unstrained in their primary shape (Figure 7c,d) or strained and fixed in a secondary shape (Figure 7a,b). A subset of films was exposed to an alternating magnetic field for 10 min at 1, 4, and 7 h, and drug release was characterized after each exposure (indicated by arrows on Figure 7a,c charts). A set of controls was run in which samples were simply incubated in PBS at 37 °C without exposure to the magnetic field (Figure 7b,d). The left side of the sample was composed of HPED TEG with 50 mg mnp and was therefore designed to release 6-MP slowly over time. The right side of the sample was composed of PPG TEG containing 100 mg mnp and was designed to rapidly release Rhod.

The strained, heat-activated samples in Figure 7b were stored at 37 °C in PBS to mimic passive release in body conditions. Minimal drug release was observed in these samples without remote actuation, indicating that drug release can be controlled with the application of the magnetic field. The unstrained samples stored at 37 °C in PBS had higher release rates (Figure 7d), showing that the use of SMPs can be used to control drug release more. In both tests that were conducted without magnetic actuation, differential release rates were observed, with a faster release of rhodamine and a slower release of 6-MP.

These trends were also seen in the magnetically actuated samples, with the highest drug release measured from unstrained samples with the application of a magnetic field (Figure 7c). The strained samples with magnetic actuation provided increased control over the release, with higher release as compared with unstrained, heated samples and lower release as compared with strained, heated samples and unstrained, magnetically actuated samples (Figure 7a).

The difference in release rates between the two sides of the polymer composite demonstrates the ability to control drug release rates. This proof of concept can be utilized in future work to achieve dual drug release of two different drugs with distinct release profiles from a single implant. The heat-activated samples at 37 °C in PBS mimic the body conditions at 37 °C and demonstrate minimal drug release without remote actuation in the strained condition.

### 3.9. Design Principles for SMP-Based Drug Delivery Devices

Initially, it was hypothesized that PPG TEG would have the slowest drug release rate due to its highest contact angle (Figure 2a) and highest wet glass transition temperature (Figure 2b). However, it was observed that PPG TEG had the fastest drug release among all the tested formulations (Figure 5a), which was attributed to its linear thermoplastic form compared with the other polymer compositions (HPED TEG and HPED PPG) that have a chemically cross-linked (thermoset) network. This result shows that the amount of cross-linking within the polymer network has a greater impact on controlled release than hydrophobicity and glass transition temperature. Cross-linked networks can physically trap drugs to a greater extent, even in samples with lower glass transition temperatures and contact angles that are plasticized and hydrated in aqueous conditions.

Magnetic particle content also had a significant impact on drug release, which can improve control compared with prior work that relies upon passive release. For example, Sivak et al., previously developed polyurethane foams for the simultaneous release of two chemotherapeutic drugs, DB-67 and Dox [31]. In that work, hydroxyl and amine groups on the drugs were allowed to react with the isocyanate groups of lysine diisocyanate methyl ester during polyurethane foam synthesis. This system depends on hydrolytic degradation of the polymeric network and subsequent release of the drugs. The materials exhibited extremely slow degradation (<0.1% as of day 70), which contributed to slow release of drugs. The fastest release rates (80 mol% release within 70 days) were obtained at 70 °C, which renders the system incompatible for biomedical applications.

Transdermal microneedles were previously used to deliver Dox in vivo across rat skin by Chen et al. [32]. This application achieved robust control over the release of doxorubicin embedded within caprolactone microneedles containing photosensitive nanomaterials. Infrared light was used to melt the microneedles and subsequently trigger drug release. This method achieved high precision over release rate via the number of on–off cycles used but is limited to transdermal drug release. The proof of concept shown in this work attains high precision over differential release rates of two drugs by tuning the polymer cross-linking, straining polymers into temporary secondary forms, altering the concentration of mnps, and applying an alternating magnetic field. This system could be applied to a similar transdermal patch or as an implanted drug depot in future work to provide on–off release of drugs. Further future work will consider the removal of the drug-delivery vehicles either surgically or via degradation after drug release is achieved.

## 4. Conclusions

This study provides a proof of concept for a unique way to tune drug delivery rates and achieve remote on-demand drug delivery from an implanted material via the application of an alternating magnetic field. The high cytocompatibility of the magnetically actuated SMPs indicates that these polymers may be suitable for future use in on-demand drug delivery. Tuning the overall polymer network provides varied mechanical and shape memory properties, which influence drug release from the materials. These scaffolds enable control over the frequency and dosage of drug delivery as required. This system could be used either to administer a single drug over multiple time points or to administer multiple drugs simultaneously. The concentration of mnps present can be used to further alter the drug delivery rate. In both the strained and unstrained conditions, the drug release amounts are three times higher with magnetic actuation compared with the same without magnetic actuation. These materials could be implanted at the target site (e.g., a carcinoma), and localized and sustained drug delivery could be triggered remotely as required to avoid multiple intravenous infusion therapies. In future work, drugs could be loaded within microspheres to achieve greater control over release rates based on alterations in the polymer chemistry and cross-link density, and the effects of magnetic field application through tissues of varying thickness can be studied to assess the potential use of this system as an implanted drug depot.

## Figures and Tables

**Table 1:** Components of synthesized polymer film compositions in wt% with catalyst amount and mixing times. HDI: hexamethylene diisocyanate, PPG: poly(propylene glycol), TEG: triethylene glycol, HPED: hydroxypropyl ethylenediamine, DBTL: dibutyl(tin) dilaurate, Dox: doxorubicin, 6-MP: 6-mercaptopurine, Rhod: rhodamine B.

Sample	HDI	PPG	TEG	HPED	DBTDL	Fe_3_O_4_ Particles	Drug (5 mg/10 g)	Mixing Time (s)
PPG TEG	29.4	50.8	19.8	0	0.8	50 mg	Dox 6-MP Rhod	15
						100 mg	Dox 6-MP Rhod	
HPED PPG	29.6	51.0	0	19.4	0.8	50 mg	Dox 6-MP Rhod	30
						100 mg	Dox 6-MP Rhod	
HPED TEG	55.8	0	22.4	21.8	0	50 mg	Dox 6-MP Rhod	60
						100 mg	Dox 6-MP Rhod	
Dual-R Dual-L	29.4 55.8	50.8 0	19.8 22.4	0 21.8	0.8 0	100 mg 50 mg	Rhod 6-MP	15 60

**Table 2:** Tensile properties of SMP films. Mean ± standard deviation displayed. *n* = 3.

Sample	Elastic Modulus (kPa)		Elongation at Break (%)		Ultimate Tensile Strength (kPa)	
	Dry	Wet	Dry	Wet	Dry	Wet
PPG TEG	80 ± 35	4 ± 2	680 ± 210	1050 ± 450	8300 ± 3200	1770 ± 120
HPED PPG	46 ± 4	21 ± 2	85 ± 9	94 ± 14	2660 ± 330	1570 ± 70
HPED TEG	595 ± 75	25 ± 2	170 ± 50	40 ± 14	29,200 ± 4000	730 ± 340

## Data Availability

The data presented in this study are available on request from the corresponding author.
